# Towards enriching and isolation of uncultivated archaea from marine sediments using a refined combination of conventional microbial cultivation methods

**DOI:** 10.1007/s42995-021-00092-0

**Published:** 2021-03-03

**Authors:** Haining Hu, Vengadesh Perumal Natarajan, Fengping Wang

**Affiliations:** 1 State Key Laboratory of Microbial Metabolism, School of Life Sciences and Biotechnology, Shanghai Jiao Tong University, Shanghai, 200240 China; 2 School of Oceanography, Shanghai Jiao Tong University, Shanghai, 200240 China

**Keywords:** Microbial cultivation, Marine sediments, Uncultivated archaea, Co-culture, *Bathyarchaeota*

## Abstract

The archaea that can be readily cultivated in the laboratory are only a small fraction of the total diversity that exists in nature. Although molecular ecology methods, such as metagenomic sequencing, can provide valuable information independent of cell cultivation, it is only through cultivation-based experiments that they may be fully characterized, both for their physiological and ecological properties. Here, we report our efforts towards enriching and isolation of uncultivated archaea from marine sediments using a refined combination of conventional microbial cultivation methods. Initially, cells were retrieved from the sediment samples through a cell extraction procedure and the sediment-free mixed cells were then divided into different size-range fractions by successive filtration through 0.8 µm, 0.6 µm and 0.2 µm membranes. Archaeal 16S rRNA gene analyses indicated noticeable retention of different archaeal groups in different fractions. For each fraction, supplementation with a variety of defined substrates (e.g., methane, sulfate, and lignin) and stepwise dilutions led to highly active enrichment cultures of several archaeal groups with *Bathyarchaeota* most prominently enriched. Finally, using a roll-bottle technique, three co-cultures consisting of *Bathyarchaeota* (subgroup-8) and a bacterial species affiliated with either *Pseudomonas* or *Glutamicibacter* were obtained. Our results demonstrate that a combination of cell extraction, size fractionation, and roll-bottle isolation methods could be a useful protocol for the successful enrichment and isolation of numerous slow-growing archaeal groups from marine sediments.

**Supplementary Information:**

The online version contains supplementary material available at 10.1007/s42995-021-00092-0.

## Introduction

The discovery of the archaea as a third domain of life on a par with Bacteria and Eukarya triggered a revolution in the field of evolutionary biology (Woese and Fox 1977; Woese et al. 1990). Following discovery of the independent evolutionary status of archaea, the study of their diversity and ecological relevance has been expanded continuously with the rapid development of culture-independent molecular ecology methods. Using molecular methods, especially the 16S rRNA gene analysis, archaea have been continuously found in various environments, not just extreme environments such as hot springs, hydrothermal vents, but also ambient environments such as soil, marine and fresh water, and sediments (DeLong 1992; Fuhrman et al. 1992; Spang et al. 2017). Recently, the rapid progress of high-throughput sequencing and analysis has facilitated the retrieval of microbial genomes directly from environments without strain isolation, thus providing the opportunity for significant advances in knowledge and understanding of the metabolic potential and ecological function of archaea in the environments (He et al. 2016; Wang et al. 2019).

In contrast to the huge contributions that culture-independent molecular techniques have made to archaeal research, archaeal cultivation techniques have made little progress (Sun et al. 2019). Most of the archaeal diversity currently referenced in public databases remains uncultivated and is known only from gene sequences obtained from molecular surveys (Schleper et al. 2005; Sun et al. 2019). There have been increasing numbers of functionally unknown and unverified genes with accumulating genomes; however, it is essentially impossible to learn new gene and pathway functions solely from sequence data. Evidently, a true understanding of the physiology of archaea and their roles in ecology requires their cultivation in the laboratory. Nevertheless, archaeal cultivation in the laboratory has thus far proven challenging, particularly for those that reside in anoxic environments with low levels of nutrients, e.g., deep-sea sediments, partly because such organisms usually have long generation times (Eme et al. 2017). Traditional techniques such as dilution to extinction and streaking on defined media are often successful for the isolation of rapidly growing microorganisms (Button et al. 1993), however, it becomes difficult for those with lower growth rates. Besides, a sudden transition from the low environmental substrate concentrations to high concentrations of standard microbial media would decrease the viability of oligotrophs, leading to incubation failure or induction of a viable but nonculturable state (VBNC) (Colwell 2000). Therefore, it is necessary to establish enrichment cultures under defined conditions, thereby facilitating studies by culture-dependent microbiological techniques combined with culture-independent approaches.

Despite these difficulties in archaeal cultivation and isolation, scientists have successfully established pure cultures or co-cultures for several groups, including *Nanoarchaeota* (Huber et al. 2002; Wurch et al. 2016), *Thaumarchaeota* (Konneke et al. 2005), *Euryarchaeota* (Sorokin et al. 2017; Zeng et al. 2009; Zhao et al. 2015), and *Lokiarchaeota* (Imachi et al. 2020). Much of the progress in expanding the range of archaea that can be grown has come from three strategies: simulation of the natural environment in vitro (de la Torre et al. 2008), co-culture with synergistic species (Park et al. 2010; Raghoebarsing et al. 2006), or refinement of selective cultivation based on genetic and transcriptional information (Wurch et al. 2016). In addition, various methods have been used to reduce the number and diversity of microbes within mixed cell samples. These include extinction–dilution whereby samples are diluted, ideally down to single cells (Konneke et al. 2005), filtration through membranes with specific pore sizes (Huber et al. 2002; Konneke et al. 2005; Sorokin et al. 2017), supplementation of specific antibiotics, such as kanamycin and streptomycin, that inhibit bacterial growth (Konneke et al. 2005; Sorokin et al. 2017), and optical tweezers—a laser-based micromanipulation technique for single-cell separation (Huber et al. 2002; Wurch et al. 2016).

Here, we tested a combination of traditional microbiological techniques, which included a cell extraction procedure, a size fractionation step, an enrichment process, and a roll-bottle isolation technique, with the goal of enriching and isolating uncultivated archaeal groups from anoxic marine sediment samples. The cell extraction procedure concentrated cells in the sediment samples as well as excluding in situ ambiguous compounds and particles. The size fractionation step sorted the sediment-free mixed cells by size using serial filtration. An enrichment process with media supplemented with potential suitable nutrients and energy sources towards targeting archaeal groups then followed. Finally, a roll-bottle technique was applied for isolating the uncultivated archaea.

## Results and discussion

### Cell extraction

Marine sediments below the sulfate-methane transition zone (SMTZ) at depth of 500–800 cm within a sediment core sampled from the Haima cold seep area on the northwest slope of the South China Sea (Niu et al. 2017) were used for archaeal isolation. The sediment samples contained around 6.4 × 10^6^ cells/g sediment as calculated by cell staining and counting (for details see Materials and methods). A cell extraction procedure was first used to separate the cells from sediment particles. Originally, this procedure was specifically devised for cell enumeration by epifluorescence microscopy (Kallmeyer et al. 2008). It was optimized in the present study for cultivation purposes by exclusion of fixatives such as formaldehyde and by applying strict anaerobic conditions during all steps in the process. After their extraction, cells were concentrated by the exclusion of interfering compounds and particles, thereby allowing downstream size fractionation as well as other operations such as high-throughput cultivation and single cell techniques.

**Fig. 1:**
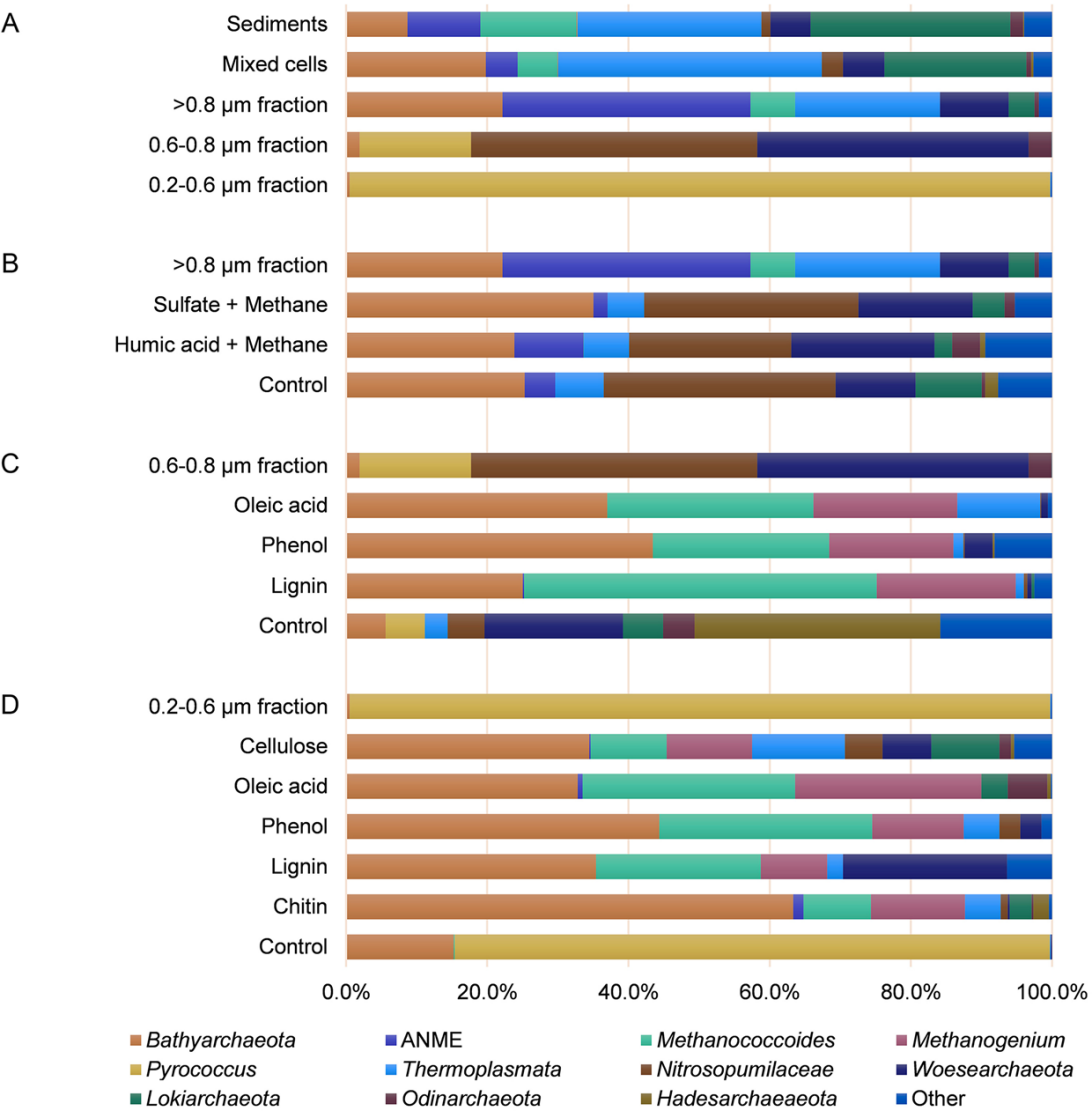
Archaeal community composition shown by 16S rRNA gene analyses. Clonal composition of the sediment samples, the mixed cells extracted from the sediment samples, and the cell-size fractions (**a**), and clonal compositions of the > 0.8 μm fraction (**b**), the 0.6–0.8 μm fraction (**c**), and the 0.2–0.6 μm fraction (**d**) after four months’ enrichment with addition of diverse substrates. ANME, anaerobic methane-oxidizing archaea

The cell extraction method retrieved around 30% of particle-free cells from the sediment samples. Archaeal taxonomic diversity was monitored both for the sediment samples and the extracted cells using archaeal 16S rRNA gene analyses. The resulting archaeal compositions before and after the cell extraction were not exactly consistent, with higher abundances of several archaeal groups like *Bathyarchaeata* and *Thermoplasmata*, along with lower levels of some other archaeal groups like ANME and *Methanococcoides* following cell extraction (Fig. 1a).

### Size fractionation

**Fig. 2:**
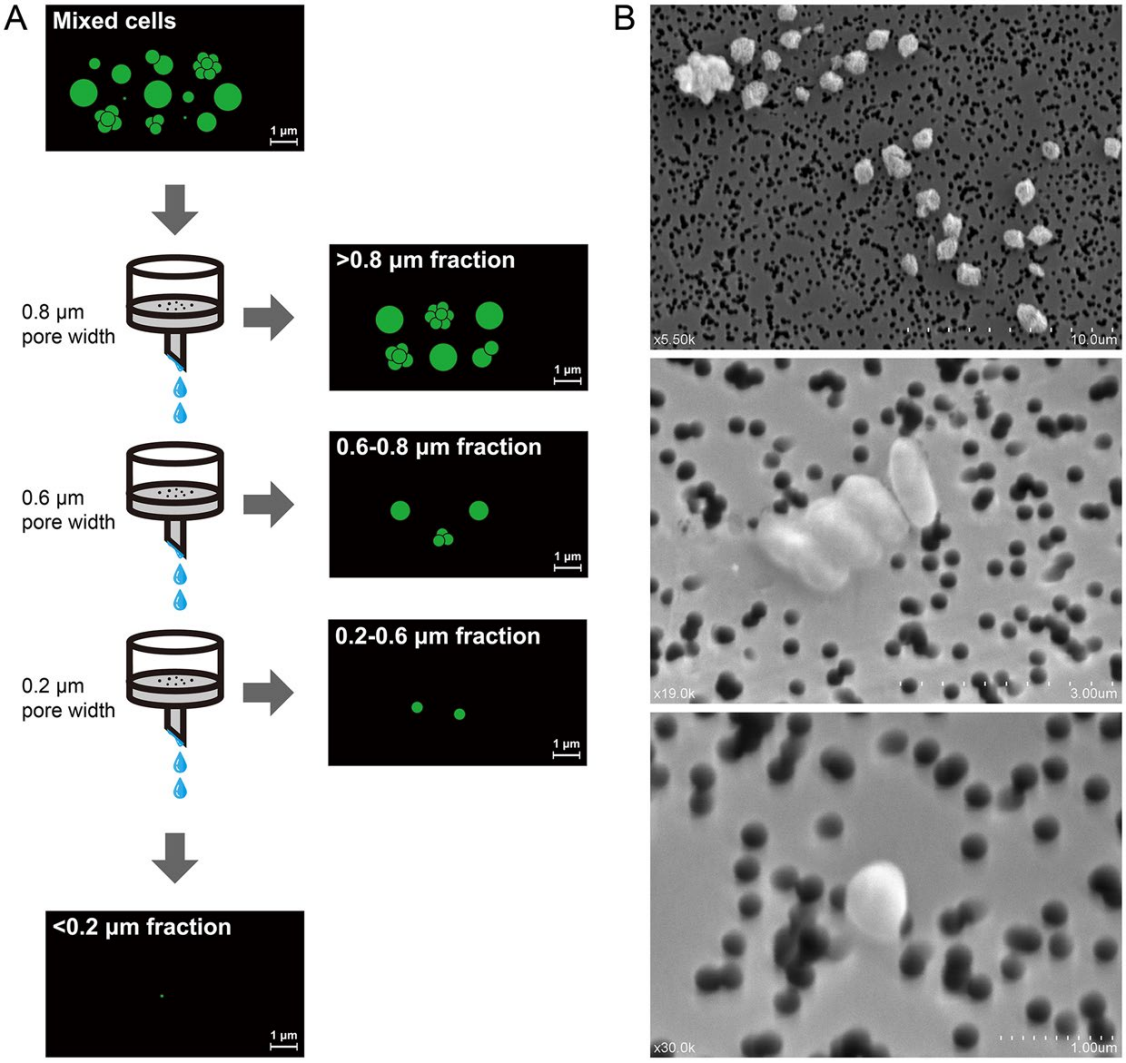
Schematic diagram of the step of size fractionation and photomicrographs of three cell fractions. **a** Schematic illustration for size fractionation. **b** Scanning electron microscopic (SEM) images of the > 0.8 μm fraction (upper), the 0.6–0.8 μm fraction (middle), and the 0.2–0.6 μm fraction (lower)

Sediment-free cells were fractionated based on their varied size ranges. To achieve this, cell suspensions were successively passed through 0.8, 0.6 and 0.2 µm pore-size membranes, leading to four cell fractions with size ranges of > 0.8 µm, 0.6–0.8 µm, 0.2–0.6 µm, < 0.2 µm, respectively (Fig. 2a). The majority of the cells (around 2.4 × 10^6^ cells/ml) were retained in the > 0.8 um fraction, followed by the 0.6–0.8 µm fraction (around 7.8 × 10^5^ cells/ml), and the 0.2–0.6 µm fraction (around 1.5 × 10^5^ cells/ml). Very few cells were seen by epifluorescence microscopy in the < 0.2 μm fraction. The cell fractions were observed by scanning electron microscopy (SEM) and showed their different size ranges, indicating that filtration through membranes with different pore sizes exerted size fractionation of the mixed cells (Fig. 2b).

Archaeal taxonomic diversity was monitored for the cell fractions using archaeal 16S rRNA gene analyses (Fig. 1a). The results showed that the size fractionation method had strong effects in decreasing the archaeal diversity and enriching specific archaeal groups. The archaeal community in the > 0.8 μm fraction was dominated by anaerobic methane-oxidizing archaea (ANME, 35.1%), followed by *Bathyarchaeota* (22.2%) and *Thermoplasmata* (20.5%). *Nitrosopumilaceae* (40.6%) and *Woesearchaeota* (38.4%) were the dominant archaeal groups in the 0.6–0.8 µm fraction. The 0.2–0.6 µm fraction was almost entirely comprised of *Pyrococcus* (99.2%).

Compared to the initial cell extracts, the relative abundance of *Bathyarchaeota* in the > 0.8 μm fraction barely changed, but a strong rise (by more than 7-fold) was observed for ANME, key players of the global carbon cycle. *Nitrosopumilaceae* and *Woesearchaeota* were enriched in the 0.6–0.8 µm fraction by more than 13-fold and more than 6-fold, respectively, and the 0.2–0.6 µm fraction increased *Pyrococcus* to predominant levels (Fig. 1a).

Various methods can be used to physically reduce the number and diversity of microbes within mixed cell samples prior to cultivation. These include filtration methods, density-gradient centrifugation or elutriation and serial dilution-to-extinction (Vartoukian et al. 2010). Filtration is one of the most common methods and has proved its simplicity and utility in several studies. For example, tiny cocci of *Nanoarchaeota* were physically isolated from their host *Ignicoccus* spheres by ultrafiltration through 0.45 μm pore-size membranes (Huber et al. 2002). Membranes with the same pore size also enabled isolation of alkaliphilic methylotrophic methanogens in pure culture in another study (Sorokin et al. 2017). Here, we adopted serial filtration with decreasing membrane pore sizes to achieve size fractionation from the mixed cells. Through this step, evident enrichment effects were achieved for ANME in the > 0.8 um fraction (Fig. 1a), which are supposed more suitable as the cultivation samples for further enrichment. Intriguingly, archaea such as *Bathyarchaeota* usually represent small-sized cells (Kubo et al. 2012), but were detained in the largest cell fraction. One of the explanations is that they might form cell aggregates either by themselves or by attachment to some other types of cells (Kubo et al. 2012). Indeed, several cell aggregates were observed in the SEM images of the > 0.8 um fraction, as shown in Fig. 2b.

### Enrichment cultures for the cell fractions

**Fig. 3:**
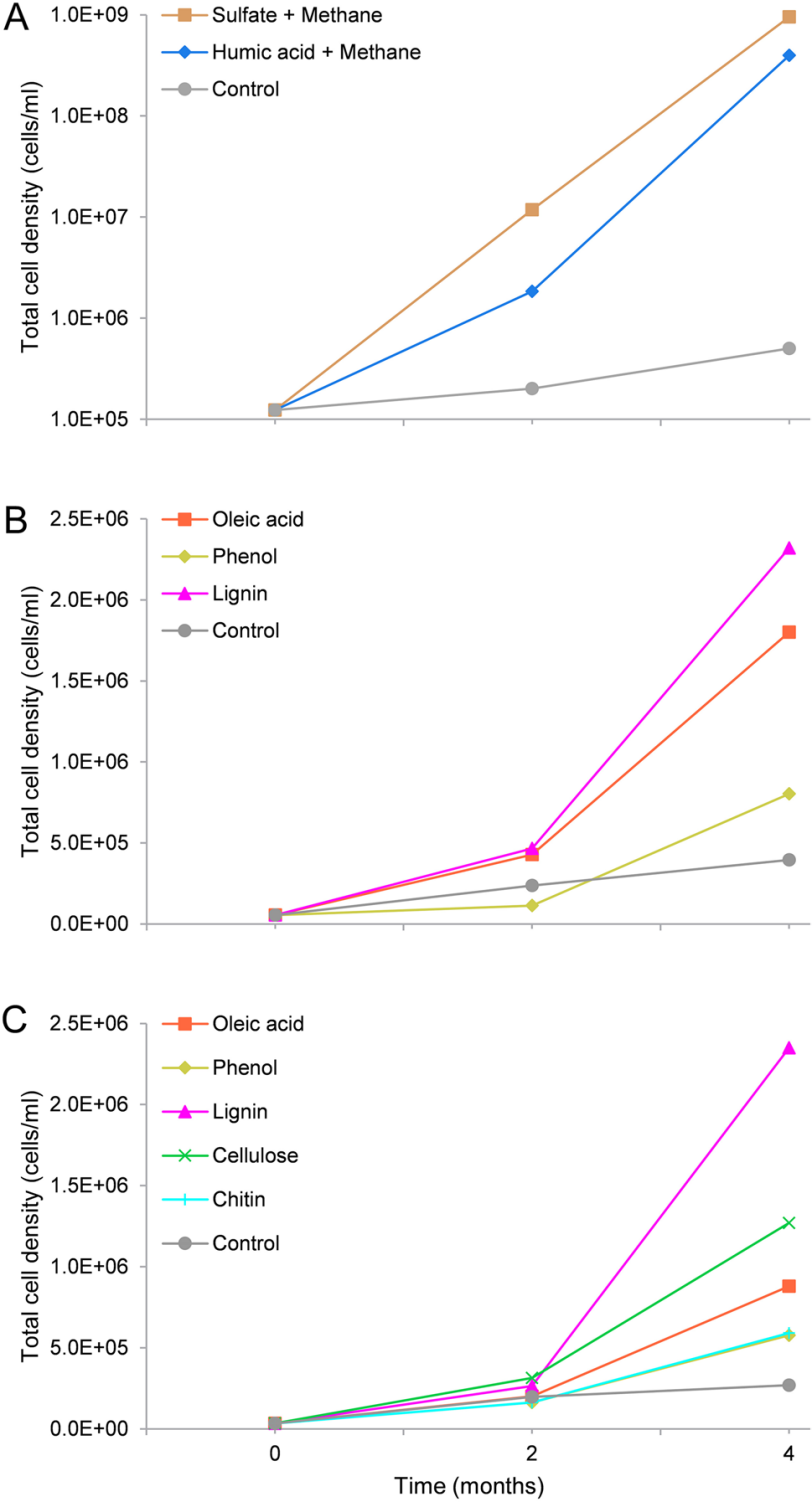
Cell counts of the > 0.8 μm fraction (**a**), the 0.6–0.8 μm fraction (**b**), and the 0.2–0.6 μm fraction (**c**) after enrichment with the addition of diverse substrates for two and four months

The two most abundant archaeal groups, *Bathyarchaeota* and ANME, in the > 0.8 μm fraction were selected as the subsequent enrichment targets for this fraction. Enrichment cultures were set up by providing a methane atmosphere as an electron donor coupled with either sulfate or humic acid as an electron acceptor. Cell fractions without the addition of these substrates were used as negative controls. Growth was monitored by cell counts and archaeal taxonomic diversity was monitored using archaeal 16S rRNA gene analyses. Total cell density in the initial inoculum was 1.2 × 10^5^ cells/ml. After four months' incubation, the numbers for both the sulfate and humic acid groups markedly increased to > 10^8^ cells/ml (Fig. 3a). Archaeal 16S rRNA gene analyses showed shifts of archaeal communities in response to different substrates (Fig. 1b). For the relative abundance of *Bathyarchaeota* within the archaeal community, the percentages of bathyarchaeotal reads remained nearly constant across the humic acid treatment, but increased slightly from 22.2 to 35.1% in the sulfate group. For ANME, no obvious enrichment was observed for any treatment.

The smaller cell fractions (i.e., the 0.6–0.8 µm and 0.2–0.6 µm fractions) were used for enrichment of potential heterotrophs. For this purpose, they were supplemented with different organic substrates that included the long-chain fatty acid oleic acid, the aromatic monomer phenol, the phenolic polymer lignin, the polymeric carbohydrate cellulose, and chitin. For the 0.6–0.8 µm fraction, the strongest growth stimulation was seen with lignin treatment. The total cell counts gradually climbed from 5.0 × 10^4^ cells/ml to > 2 × 10^6^ cells/ml following four months of incubation (Fig. 3b). Likewise, the lignin treatment supported the maximum growth for the 0.2–0.6 µm fraction, exerting a far better stimulation effect than other organic substrates (Fig. 3c). Archaeal diversity analyses showed that the relative abundance of *Bathyarchaeota* significantly increased 13- to 23-fold after amendment with diverse organic substrates for the 0.6–0.8 µm fraction (Fig. 1c). Great increases of bathyarchaeotal abundance were also seen for the 0.2–0.6 µm fraction, from 0.5% to over 30% (Fig. 1d). Within the bathyarchaeotal group, all amplicon sequence variants (ASVs) aligned were subject to phylogenetic analyses to determine their closest phylogenetic affiliations. The results showed that *Bathyarchaeota* subgroup-8 became dominant, accounting for 41.2–96.3% of all bathyarchaeotal 16S rRNA gene reads (Supplementary Table S1).

*Bathyarchaeota* is widespread in a variety of habitats, including terrestrial, freshwater, hypersaline, and hydrothermal, environments (Kubo et al. 2012) and is one of the most abundant groups in marine sedimentary archaeal communities (He et al. 2016; Lloyd et al. 2013). In the last two decades, physiological and genomic evidence have identified this ubiquitous phylum at a key player in global carbon biogeochemical cycling (Feng et al. 2019; Fillol et al. 2016; He et al. 2016; Kubo et al. 2012; Lloyd et al. 2013). Metagenomic profiling and stable isotope probing have indicated the potential of *Bathyarchaeota* to anaerobically utilize detrital proteins, polymeric carbohydrates, fatty acids, aromatic compounds, alkanes, and/or potentially other organic matter (Feng et al. 2019; He et al. 2016; Wang et al. 2019; Zhou et al. 2018).

*Bathyarchaeota* has been detected as one of the dominant archaeal groups in the sediments of the South China Sea, and is considered to be heterotrophic (Yu et al. 2017). For the 0.6–0.8 µm and 0.2–0.6 µm fractions, in the present study, *Bathyarchaeota* was significantly enriched with addition of different organic substrates (Fig. 1c, d). A recent work by Yu et al. (2018) demonstrated that lignin stimulated the growth of Bathy-8 subgroup, and it was concluded that they were capable of assimilating CO_2_ autotrophically while utilizing lignin as an energy source (Yu et al. 2018). The stimulation of the growth of Bathy-8 by lignin in this study is not surprising, but it is noteworthy that the sediment samples we used were from deep-sea sediments and the cultivation temperature was ~ 10 °C. Therefore, we anticipate that the Bathy-8 cells enriched here are a low-temperature-adapted group, in contrast to those isolated by Yu et al. (2018) which were from a coastal environment and were incubated at 30 °C. Nevertheless, although it seems likely that lignin-utilizing capability is a common trait in the Bathy-8 subgroup, further investigations are needed to understand the role of lignin in the growth of *Bathyarchaeota* and to clarify the mechanisms involved in the metabolism of lignin by *Bathyarchaeota*.

### Archaeal isolation using a roll-bottle technique

**Fig. 4:**
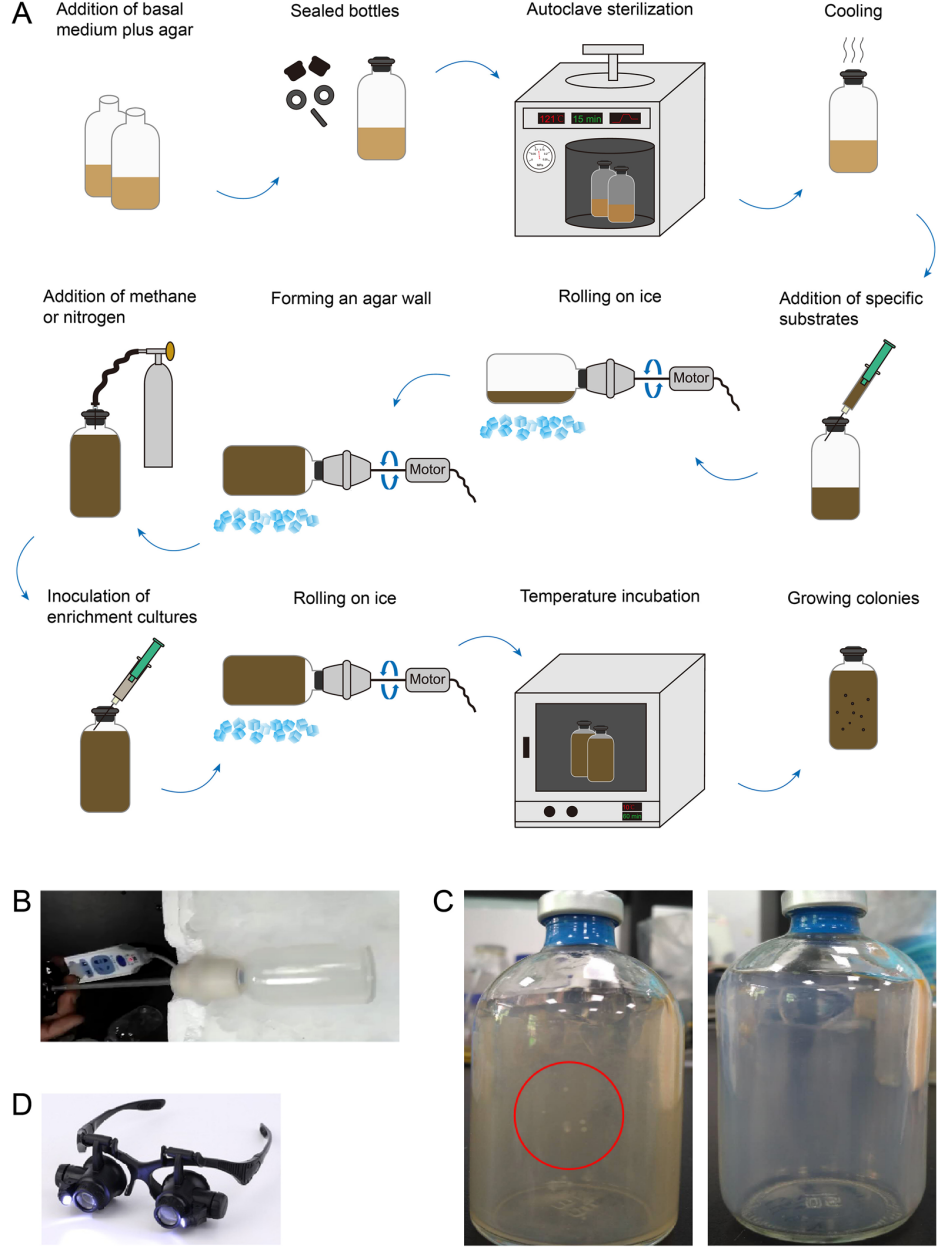
Schematic diagram and photographs of the roll-bottle technique. **a** Scheme illustrating the preparation of roll-bottles. **b** A rolling bottle on ice. **c** A well-prepared roll-bottle with media containing lignin (left), and another one with media containing sulfate plus methane (right). **d** 10 × or 20 × magnifying lenses. Colonies present in the lignin-amended roll-bottles (**c** left, shown in the red circle) were visible to the naked eye, whereas those in the sulfate/methane-amended roll-bottles (**c** right) were very small and thus had to be observed and picked with the help of 10 × or 20 × magnifying lenses (**d**)

We attempted different methods to further enrich and isolate *Bathyarchaeota* using methods including streaking/spreading on agar plates, dilution to extinction with microtiter plates, and micro-droplet assays, but all failed due to the difficulties in maintaining anaerobic and long-term culture conditions (data not shown). To overcome these difficulties, we adopted and modified a roll-bottle technique based on the Hungate method for anaerobic incubation (Balch and Wolfe 1976; Hungate 1950, 1969; Ljungdahl 1986). These roll-bottles enable isolation methods, such as streaking/spreading on defined media, to be carried out in tightly sealed bottles, which provide a stable growing environment, strict anaerobic conditions, and little risk of bio-contamination from the surroundings (Fig. 4a, b). The culture conditions in the roll-bottles were identical to those used in the enrichment process for each cell fraction except for the addition of 1.5% agar. After four months’ incubation, colonies were formed on the agar walls, and a total of 90 colonies were picked from the roll-bottles. These included 10, 30 and 50 colonies, respectively, from the chitin-, the lignin-, and the sulfate/methane-amended roll-bottles. Colonies present in the lignin-amended roll-bottles were visible to the naked eye, whereas those in the chitin- and sulfate/methane-amended roll-bottles were very small (Fig. 4c) and had to be observed and picked with the help of 10 × or 20 × magnifying lenses (Fig. 4d). In contrast, the cell fractions directly used for the roll-bottle technique yielded no colonies, which may result from their much lower initial cell abundances, compared to the high cell densities of enrichment cultures.

Recovered colonies were transferred into liquid media with the same substrates and under the same atmospheric conditions for further cultivation and screening purposes. After four weeks of incubation, a total of ten cultures were randomly selected for PCR amplification using archaeal and bacterial 16S rRNA gene-specific primers. The PCR results showed that the archaeal primers yielded positive amplifications for three cultures (Table 1). These archaeal amplicons were sequenced, followed by phylogenetic analyses to determine the closest phylogenetic affiliations. Phylogenetic analyses indicated that these archaea were most closely related to *Bathyarchaeota* (subgroup-8), and comprised a parallel lineage with several dominant Bathy-8-related ASVs (Supplementary Fig. S1a). Meanwhile, their bacterial counterparts were identified as *Pseudomonas* sp. or *Glutamicibacter* sp. (Supplementary Fig. S1b, c). The three co-cultures were designated as 5cc, 7cc and 8cc. Based on quantitative PCR (qPCR) results for these co-cultures, the bacterial and archaeal 16S rRNA gene copy numbers were approximately 10^8^ and 10^6^ copies/ml, respectively. The co-cultures were also confirmed by fluorescence in situ hybridization (FISH) using bacterial and archaeal 16S rRNA-targeted probes (Supplementary Fig. S2).

**Table 1:** Colonies randomly selected from the roll-bottles for PCR amplification using archaeal and bacterial 16S rRNA gene-specific primers

Colony	Roll-bottle	PCR amplification		Co-culture	Co-culture name
		Archaeal (21F and 958R^a^)	Bacterial (27F and 1492R^b^)		
1	Lignin	N^c^	A^d^	–	
2	Lignin	N	A	–	
3	Lignin	N	A	–	
4	Lignin	N	A	–	
5	Lignin	A	A	*Bathyarchaeota* (subgroup-8) and *Pseudomonas* sp.	5cc
6	Lignin	N	A	–	
7	Sulfate/methane	A	A	*Bathyarchaeota* (subgroup-8) and *Pseudomonas* sp.	7cc
8	Sulfate/methane	A	A	*Bathyarchaeota* (subgroup-8) and *Glutamicibacter* sp.	8cc
9	Chitin	N	A	–	
10	Chitin	N	A	–	

^a^Archaeal 16S rRNA gene-specific primer sets

^b^Bacterial 16S rRNA gene-specific primer sets

^c^No amplification by PCR

^d^Amplified by PCR

Within microbial communities, complex cell–cell communications are crucial for some microorganisms since individual species often lack the ability to produce all essential factors necessary for survival. The exchange between these microorganisms includes secondary metabolites, growth factors, signaling molecules, and even electron (Meyerdierks et al. 2010; Sun et al. 2019). Hence, co-culture with synergistic species is an effective solution for growing fastidious microorganisms (Park et al. 2010; Raghoebarsing et al. 2006).

For instance, van de Pas-Schoonen et al. (2009) first reported cultivation and isolation of hydrogenotrophic methanogens using a co-culture method, in which a heterotrophic H_2_-producing bacterium was used as a helper organism. Another example is Direct Interspecies Electron Transfer (DIET), a possible mode of shuttling electrons from ANME to a sulfate-reducing partner, which facilitates their cooperative growth (McGlynn et al. 2015; Meulepas et al. 2009). In the present study, the three co-cultures derived from the lignin- and sulfate/methane-amended roll-bottles consisted of *Bathyarchaeota* (Subgroup-8) and a bacterial species affiliated with ether *Pseudomonas* or *Glutamiciabacter* (Table 1). The results suggested the ability of the three co-cultures to utilize lignin or sulfate/methane for growth. However, whether there is syntrophic association between *Bathyarchaeota* and the bacterial members is still ambiguous and remains to be investigated.

For further isolation of *Bathyarchaeota* in pure culture from the three co-cultures, bacterial growth should be restricted as much as possible. To attain this, we adopted different methods, such as addition of diverse antibiotics and provision of autotrophic conditions (NaHCO_3_/H_2_). Although antibiotics are commonly used in archaeal isolation work and are of great help in some cases (Imachi et al. 2020; Simon et al. 2005; Sorokin et al. 2017), they exhibited unsatisfactory effects in stopping bacterial growth in our study (Supplementary Table S2). One explanation for their poor performances is that in low-temperature sediments, secondary metabolites produced by bacteria are stable for a long time, thus enabling these inhabitants to be naturally resistant to diverse antibiotics (Chen and Zhou 2014; Li et al. 2012; Xu et al. 2009).

Some lineages of *Bathyarchaeota* are believed to be acetogens, being capable of homoacetogenesis, a metabolism so far restricted to the domain Bacteria (He et al. 2016). He et al. (2016) identified bathyarchaeotal genomic bins that had the genetic potential for inorganic carbon fixation via the reductive acetyl-CoA (Wood–Ljungdahl, WL) pathway. Hence, autotrophic conditions (NaHCO_3_/H_2_) were provided here to favor the growth of *Bathyarchaeota* as well as to inhibit bacterial growth. The qPCR results showed that bathyarchaeotal 16S rRNA gene copy numbers in the three co-cultures increased tens- to hundreds-fold after one month of incubation (Supplementary Fig. S3a). Intriguingly, the bacterial counterparts were also growing well with total cell density climbing gradually from 10^5^ to over 10^8^ cells/ml (Supplementary Fig. S3b). To date, there is no direct evidence for the autotrophic ability of pure *Pseudomonas* or *Glutamiciabacter* cultures. Therefore, the two bacterial species in the co-cultures are also worthy of in-depth study along with the target archaea. Given that both *Pseudomonas* sp. and *Glutamiciabacter* sp. represented an overwhelming predominance of abundance over their archaeal partners in the co-cultures, it will be much easier to isolate the two bacterial members from the co-cultures. Hence, isolation of *Pseudomonas* sp. and *Glutamiciabacter* sp. in pure culture will be another priority of subsequent work, followed by whole genome sequencing and bioinformatic analyses, alongside the efforts for isolation of *Bathyarchaeota*.

In conclusion, we report our efforts towards enriching and isolating uncultivated archaea from marine sediments using a refined combination of conventional microbial cultivation methods. These methods include cell extraction, size fractionation, enrichment, and isolation, which enabled us to finally obtain three co-cultures composed of *Bathyarchaeota* (subgroup-8) and two bacterial species. Our results suggest that combining these conventional methods in a particular way could facilitate the successful enrichment and isolation of numerous slowly growing archaeal groups from marine sediments.

## Materials and methods

### Sediment sampling

A sediment core (QDN-14B, 830 cm) was collected from the Haima cold seep area on the northwest slope of the South China Sea during the R/V Haiyang IV cruise on April 2, 2015 (Niu et al. 2017). The sampling location was ∼ 600 m east of the ROV1 site at approximately 1380 m water depth. Previous studies using culture-independent molecular analyses (i.e., 16S rRNA gene tag-sequencing) showed that *Bathyarchaeota* accounted for 7–36% of the total archaeal groups in the QDN-14B core (Niu et al. 2017). In this study, we used the sediments below the sulfate–methane transition zone (SMTZ) at a depth of 500–800 cm within the sediment core. The sediment samples were preserved anaerobically at 4 °C in the dark until enrichment cultures were initiated.

### Cell extraction

Cells were separated from the sediment samples in several steps following the methodology of Kallmeyer et al. (2008). Specifically, 0.5 g of each sediment sample was thoroughly mixed with 750 µl artificial synthetic seawater (23.5 g NaCl, 5 g MgCl_2_, 0.55 g KCl, 0.08 g KBr, 1.1 g CaCl_2_, 0.025 g Boric acid, 0.0225 g SrCl_2_, 0.003 g NaF in 1 L sterile water) by vortexing slowly for up to 10 min. The sediment slurry was centrifuged at 2000 × *g* for 5 min, and the clean supernatant was collected. The pellet was resuspended with 1 ml of acetate buffer containing 5 ml/L (0.5% vol/vol) acetic acid and 35 g/L (0.6 mol/L) NaCl, and vortexed at low speed for 10 min. After the complete dissolution of carbonate minerals, the carbonate-free sample was centrifuged at 2000 × *g* for 5 min, and the clean supernatant was removed and retained. The pellet was diluted with 900 µl NaCl solution (35 g/L (0.6 mol/L) NaCl), and 100 µl detergent mix used for hydrolyzing extracellular polymers was added. The detergent mix contained 146.1 g/L (0.5 mol/L) EDTA, 133 g/L (0.5 mol/L) sodium pyrophosphate, 5 ml/L (0.5% vol/vol) Tween 80 and 35 g/L (0.6 mol/L) NaCl. After vortexing gently for 20 min, a cushion of 500 µl 50% (wt/vol) Nycodenz was layered below the mixture with a syringe and a 12-gauge needle. The mixture was then centrifuged at 2000 × *g* for 5 min. The supernatant was transferred and retained and the remaining Nycodenz was discarded. The pellet was resuspended with 1 ml artificial synthetic seawater, followed by gently vortexing for 20 min. Density gradient centrifugation with addition of Nycodenz was repeated as described above. The supernatant collected from this round of density separation as well as those from the preceding treatments were pooled and used for further processing. All the reagents were filtered through 0.1 µm filter (Millipore) to exclude contamination before use.

### Size fractionation

The cells recovered from the sediments were fractionated based on their different size ranges. To attain this, polycarbonate filters with 0.8, 0.6 and 0.2 µm pore sizes (GTTP; Millipore) were used. The cell suspensions were successively passed through the 0.8, 0.6 and 0.2 µm filters (Fig. 2a). To decrease the probability of cell breakage and clogging, the filtration was performed using a vacuum pump at a low pressure, and the volumes filtered were constrained to less than 1 ml. Retentates were recovered by washing with artificial synthetic seawater three times. Consequently, four cell fractions with size ranges at > 0.8 µm, 0.6–0.8 µm, 0.2–0.6 µm, < 0.2 µm were obtained.

### Enrichment cultures

Enrichment for each cell fraction was performed in 100 ml serum vials containing 50 ml media and sealed with butyl rubber stoppers and aluminum crimp seals. The basal medium was artificial synthetic seawater (as described above), supplemented with 1 ml nonchelated trace element mixture, 1 ml vitamin solution (Widdel and Bak 1998), and finally reduced by the addition of 1 mmol/L Na_2_S. The > 0.8 μm fraction was incubated under an atmosphere of 99.99% methane gas and with addition of 28 mmol/L of sodium sulfate or 0.05% of humic acid. The incubation for the 0.6–0.8 µm and 0.2–0.6 µm fractions was carried out under an atmosphere of 99.99% N_2_ gas and supplemented with one of the following five organic growth substrates at a dose of 50 mg/L: oleic acid, phenol, lignin, cellulose, and chitin. Additionally, for each fraction, 5 mmol/L of NaHCO_3_ was added as an inorganic carbon source. Initially, a volume of 5 ml of each cell fraction was inoculated into each medium, followed by incubation at 10 ℃ without shaking in the dark for four months. The culture vessels were set up in duplicate for each substrate. Meanwhile, each cell fraction was also inoculated into basal medium without the addition of any substrates and incubated under the same conditions as controls. Growth was monitored by cell counts in the duplicate cultures, and repeated at least twice. When total cell counts stopped increasing, the enrichment cultures were diluted by one or two serial 1:100 transfers. Archaeal taxonomic diversity was monitored using archaeal 16S rRNA gene analyses.

### Cell enumeration

Cell enumeration was conducted following established protocols (Morono et al. 2009). Particularly, 200 µl of cell suspensions or liquid cultures were mixed with 5 ml NaCl/formaldehyde solution [35 g/L (0.6 mol/L) NaCl and 4% (wt/vol) formaldehyde] and filtered using 0.2 μm polycarbonate GTTP membranes (Millipore). The cells retained on the filter membranes were stained with SYBR Green I and were counted using epifluorescence microscopy using a blue filter set. For cell counting of the < 0.2 µm fraction, 500 µl of cell suspensions were used instead of the routine volume. For the sediment samples, a 0.002 g subsample was diluted with 200 µl NaCl/formaldehyde solution and used for cell counting as described above.

### Nucleic acid extraction and archaeal 16S rRNA analysis

DNA extraction was performed as described previously (Natarajan et al. 2016). For PCR amplification of the hypervariable V4 regions of archaeal 16S rRNA genes, we used the multi-tag primer sets U519F and Arch806R (Supplementary Table S3). Each 50 µl PCR mixture contained 10 × PCR buffer, dNTP (100 μmol/L each), 0.25 μmol/L of each primer, 2.5 U of DNA polymerase (Ex-Taq; TaKaRa), and 6–10 ng of total DNA. The PCR conditions were as follows: initial denaturation at 95 ℃ for 3 min; 35 cycles of denaturation at 94 °C for 40 s, annealing at 56 °C for 1 min, and extension at 72 °C for 1 min; and a final extension at 72 °C for 10 min. Amplicons were purified using an E.Z.N.A. Gel Extraction Kit (Omega Bio-Tek) according to the manufacturer’s instructions and sequenced on the MiSeq platform (Illumina, USA).

Sequences of Illumina sequencing raw data were analyzed with QIIME 2 2020.11 (Bolyen et al. 2019). Demultiplexed raw sequence data were quality-filtered and denoised with DADA2. All amplicon sequence variants (ASVs) were aligned with MAFFT and used to construct a phylogeny with FastTree 2.1.11. Taxonomy was assigned to ASVs using the q2-feature-classifier classify-sklearn naïve Bayes taxonomy classifier against Silva 16S rRNA RefSeq Version 132 reference sequences. Relative abundances were estimated after samples were subsampled without replacement (rarefied) to 9160 sequences per sample. ASVs affiliated with *Bathyarchaeota* were subject to further phylogenetic analyses using bathyarchaeotal 16S rRNA reference sequences from Zhou et al. (2018) as phylogenetic anchors. The sequences were aligned with MAFFT, followed by construction of a phylogeny with FastTree 2.1.11.

### A roll-bottle technique for archaeal isolation

Archaeal isolation from the enrichment cultures was conducted by conventional methods including streaking/spreading on agar plates, dilution to extinction with microtiter plates, and micro-droplet assays, as well as the roll-bottle technique developed in this study. The roll-bottles were prepared as shown in Fig. 4a. In brief, 100 ml serum vials containing 20 ml media were sealed with butyl rubber stoppers and aluminum crimp seals. Apart from supplementation of 1.5% agar, the media and the headspace atmosphere were identical to those used in the enrichment process for each cell fraction. After autoclave sterilization, the vials were cooled by rapid rotation on ice until the media became solid, forming a layer of uniform thickness on the inside wall (Fig. 4b). The serial dilutions (10^–1^–10^–10^) of the enrichment cultures were prepared and for each dilution a 150 µl aliquot was inoculated into a vial. The vial was then rapidly rotated to make the cells spread uniformly onto the media layer. Vials were incubated vertically at 10 °C in the dark until colonies were observed with a pair of 10 × or 20 × magnifying spectacles (Fig. 4c, d). Meanwhile, cell fractions without the enrichment process were directly inoculated into roll-bottles with the same media and incubated under the same conditions as controls.

Colonies were picked and transferred into liquid media with the same substrates under the same atmospheric conditions for further cultivation and screening purposes. The bacterial and archaeal community compositions were confirmed by PCR amplification of 16S rRNA genes using primer sets 27F/1492R and Arch21F/Arch958R, respectively. The amplicons were purified and sequenced on the Sanger platform. Bacterial and archaeal abundances of the co-cultures were confirmed by quantitative PCR (qPCR).

### Phylogenetic analyses

To identify relevant gene homologues of the amplified archaeal and bacterial 16S rRNA genes, reference sequences were collected from previous publications (Kubo et al. 2012; Zhou et al. 2018) and the NCBI database (https://www.ncbi.nlm.nih.gov/), respectively. To construct phylogenetic trees, the sequences were aligned with ClustalW. Aligned sequences were subjected to a Neighbor-joining analysis implemented in the molecular evolutionary genetics analysis (MEGA) program, version 7 (Kumar et al. 2016), with the Maximum Composite Likelihood model. Bootstrap values are based on 1000 replicates.

### Further isolation of *Bathyarchaeota* in pure culture

To further isolate *Bathyarchaeota* in pure culture from the co-cultures, treatments with different antibiotics (e.g., Vancomycin, Chloramphenicol, Kanamycin, Ampicillin and Rifampicin) with varied concentrations (3–50 μg/ml) (Supplementary Table S2) and autotrophic conditions (10 mmol/L NaHCO_3_ in the media and 99.99% H_2_ gas in the headspace) were employed to inhibit bacterial growth. Growth was monitored by cell counts. Bathyarchaeotal abundance was monitored by qPCR.

### Quantification of bacterial, archaeal and bathyarchaeotal 16S rRNA genes

For bacterial, archaeal, and bathyarchaeotal 16S rRNA gene amplifications, the primer sets Bac341F/prokaryotic519R, Uni519F/Arch908R, and Bathy-442F/Bathy-644R, respectively, were used (Supplementary Table S3). The reaction mixture (20 µl) included 10 µl SYBR Premix Ex Taq, 0.4 µl 50 × ROX reference dye, 0.8 μmol/L each forward and reverse primer, and 1 µl template DNA. More details, such as primer sequences and PCR conditions, are presented in Supplementary Table S3. For construction of standard curves for the primer set Bac341F/prokaryotic519R, we used dilution series of 16S rRNA gene fragments of *Escherichia coli*, which was obtained using the bacterial 16S rRNA gene-specific primers 27F/1492R (Weisburg et al. 1991). The standard curves for the primer sets Uni519F/Arch908R and Bathy-442F/Bathy-644R were constructed with dilution series of bathyarchaeotal 16S rRNA gene fragments amplified using the archaeal 16S rRNA gene-specific primers Arch21F/Arch958R (DeLong 1992) and the bathyarchaeotal 16S rRNA gene-specific primers Bathy-442F/Bathy-644R (Yu et al. 2017), respectively. The R^2^ and amplification efficiency of each individual qPCR assay are shown in Supplementary Table S3. All assays were performed in triplicate.

### Fluorescence in situ hybridization

Cells were fixed with 4% paraformaldehyde in artificial synthetic seawater for 6 h at 4 °C and stored in 50% ethanol with phosphate-buffered saline (PBS; 130 mmol/L NaCl, 10.8 mmol/L Na_2_HPO_4_, 4.2 mmol/L NaH_2_PO_4_ [pH 7.2]) at − 20 °C. FISH was performed on cells filtered onto 0.2 μm polycarbonate GTTP membranes (Millipore) based on a method described previously (Pernthaler et al. 2001). For detection of bacteria and archaea, we used a bacteria-specific probe EUB338 (Amann et al. 1990), and an archaea-specific probe ARC915 (Stahl and Amann 1991), respectively. The oligonucleotide probes EUB338 and ARC915 were labelled by Alexa Fluor 594 and Alexa Fluor 488, respectively. After hybridization, the cells were counterstained with 5 μg/ml DAPI for 10 min at room temperature to visualize the cells by microscopy. Observation of the FISH samples was carried out using an epifluorescence microscope (Nikon ECLIPSE 90i, Tokyo, Japan) coupled with an illuminator (Nikon INTENSILIGHT C-HGFIE, Tokyo, Japan), and appropriate filter sets for Alexa Fluor 594, Alexa Fluor 488 and DAPI fluorescence.

### Scanning electron microscopy

Cell suspensions were fixed with 3% glutaraldehyde in PBS (pH 7.2) for 1 h at room temperature. After fixation, cells were washed with PBS three times and then dehydrated in a graded ethanol series (30%, 50%, 75%, 90% and 2 × 100%) for 5 min each. Cells were then filtered onto 0.2 μm polycarbonate GTTP membranes (Millipore), and critically point dried with L-CO_2_. Each membrane was mounted on an aluminum stub, sputter-coated with gold, and examined with a scanning electron microscope (Hitachi S3400N, Tokyo, Japan).

## Supplementary Information

Below is the link to the electronic supplementary material.

Supplementary file1 (DOCX 611 KB)

## Data Availability

Illumina RNA-seq datasets have been deposited in the GenBank database (accession no. PRJNA688025). 16S rRNA gene sequences amplified from the co-cultures have been deposited in the GenBank database (accession no. MW407026, MW407027, MW407028, MW407029, MW407030, and MW407031).
