# Supplementary material for: Towards enriching and isolation of uncultivated archaea from marine sediments using a refined combination of conventional microbial cultivation methods
Source: Mar Life Sci Technol. 2021 Mar 3;3(2):231–42. doi: 10.1007/s42995-021-00092-0 (PMC10077295; doi:10.1007/s42995-021-00092-0)
Supplement: Supplementary file 1 — Supplementary file1 (DOCX 611 KB) [file 42995_2021_92_MOESM1_ESM.docx]

|  |  | 0.6-0.8 μm fraction | | |  | 0.2-0.6 μm fraction | | | | |
| --- | --- | --- | --- | --- | --- | --- | --- | --- | --- | --- |
| Subgroup |  | Oleic acid (%) | Phenol (%) | Lignin (%) |  | Cellulose (%) | Oleic acid (%) | Phenol (%) | Lignin (%) | Chitin (%) |
| Bathy-8 |  | 51.6 | 87.1 | 86.9 |  | 41.2 | 86.7 | 77.8 | 78.5 | 96.3 |
| Bathy-17 |  | 27.1 | 0.2 | 0.2 |  | 10.3 | 6.1 | 0.0 | 8.6 | 0.0 |
| Bathy-15 |  | 0.0 | 1.4 | 0.8 |  | 10.2 | 0.7 | 0.0 | 12.4 | 0.0 |
| Bathy-3 |  | 0.7 | 0.0 | 1.1 |  | 13.7 | 4.6 | 6.5 | 0.0 | 1.5 |
| Bathy-6 |  | 0.0 | 7.7 | 0.0 |  | 7.2 | 0.0 | 0.0 | 0.0 | 0.0 |
| Bathy-12 |  | 7.5 | 1.3 | 1.2 |  | 3.6 | 0.1 | 0.3 | 0.0 | 0.8 |
| Bathy-2 |  | 0.0 | 0.0 | 0.0 |  | 4.2 | 0.3 | 7.8 | 0.4 | 0.0 |
| Other |  | 13.1 | 2.3 | 9.7 |  | 9.6 | 1.6 | 7.7 | 0.0 | 1.5 |

**Supplementary Table S1** Percentage of sequences affiliated with different bathyarchaeotal subgroups in total bathyarchaeotal sequences

**Supplementary Table S2** Antibiotic treatments of the co-cultures to restrict bacterial growth

| Antibiotics | Concentration (μg/ml) | Bacteria | Archaea |
| --- | --- | --- | --- |
| Vancomycin | 3 | —^a^ | — |
|  | 10 | — | — |
|  | 30 | +^b^ | + |
| Chloramphenicol | 10 | — | — |
|  | 25 | — | — |
| Kanamycin | 50 | — | — |
| Ampicillin | 50 | — | — |
| Rifampicin | 10 | — | — |

^a^No obvious growth inhibition

^b^Slight growth inhibition

**Supplementary Table S3** Primers and conditions used in PCR and qPCR

| Target description | Primer name | Sequence (5′–3′) | PCR type | Annealing temp (℃) | Product length (bp) | Ref. | R^2^ | Efficiency (%) |  |
| --- | --- | --- | --- | --- | --- | --- | --- | --- | --- |
| Archaea | U519F | XXXXXXXXXXX^a^-YMGCCRCGGKAAHACC | PCR | 56 | 287 | Song et al. (2013) |  |  |  |
|  | Arch806R | GGACTACNSGGGTMTCTAAT |  |  |  |  |  |  |  |
| Bacteria | Bac341F | CCTACGGGWGGCWGCA | qPCR | 56 | 389 | Jorgensen et al. (2012) | 0.99 | 100 |  |
|  | Prokaryotic519R | TTACCGCGGCKGCTG |  |  |  | Ovreås et al. (1997) |  |  |  |
| Archaea | Uni519F | CAGCMGCCGCGGTAA | qPCR | 60 | 178 | Ovreås et al. (1997) | 0.99 | 96 |  |
|  | Arch908R | CCCGCCAATTCCTTTAAGTT |  |  |  | Jorgensen et al. (2012) |  |  |  |
| *Bathyarchaeota* | Bathy-442F | AGACTGGTGTCAGCCGCC | qPCR | 58 | 202 | Yu et al. (2017) | 0.99 | 86 |  |
|  | | Bathy-644R | TCGCCACTGGTGGTCCTC |  |  |  |  |  |  |

^a^Various key tags for each sample

Jorgensen SL, Hannisdal B, Lanzen A et al. (2012) Correlating microbial community profiles with geochemical data in highly stratified sediments from the Arctic Mid-Ocean Ridge. Proc Natl Acad Sci U S A 109:E2846-E2855

Ovreås L, Forney L, Daae FL, Torsvik V (1997) Distribution of bacterioplankton in meromictic Lake Saelenvannet, as determined by denaturing gradient gel electrophoresis of PCR-amplified gene fragments coding for 16S rRNA. Appl Environ Microbiol 63:3367-3373

Song ZQ, Wang L, Wang FP et al. (2013) Abundance and diversity of archaeal accA gene in hot springs in Yunnan Province, China. Extremophiles 17:871-879

Yu T, Liang Q, Niu M, Wang F (2017) High occurrence of *Bathyarchaeota* (MCG) in the deep-sea sediments of South China Sea quantified using newly designed PCR primers. Environ Microbiol Rep 9:374-382


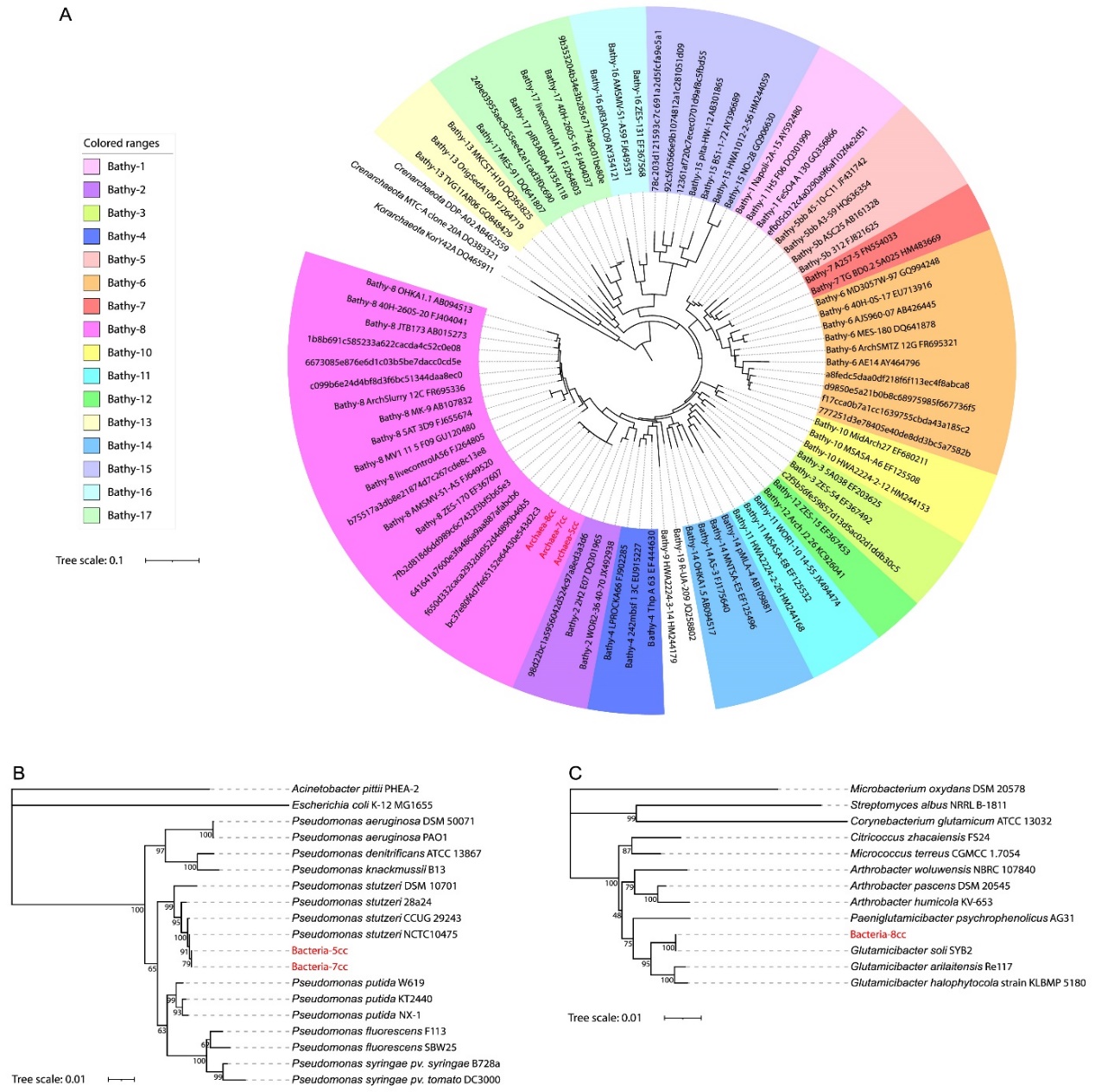


B

**Supplementary Fig. S1** Neighbor-joining phylogenetic trees of archaeal and bacterial amplicons of the co-cultures (marked in red), and the ASVs sequences (marked in bold). **a** The 20 most dominant ASVs sequences in total bathyarchaeotal sequences, and the archaeal amplicons of the co-culture 5cc, 7cc and 8cc. **b** The bacterial amplicons of the co-culture 5cc and 7cc. **c** The bacterial amplicon of the co-culture 8cc

**
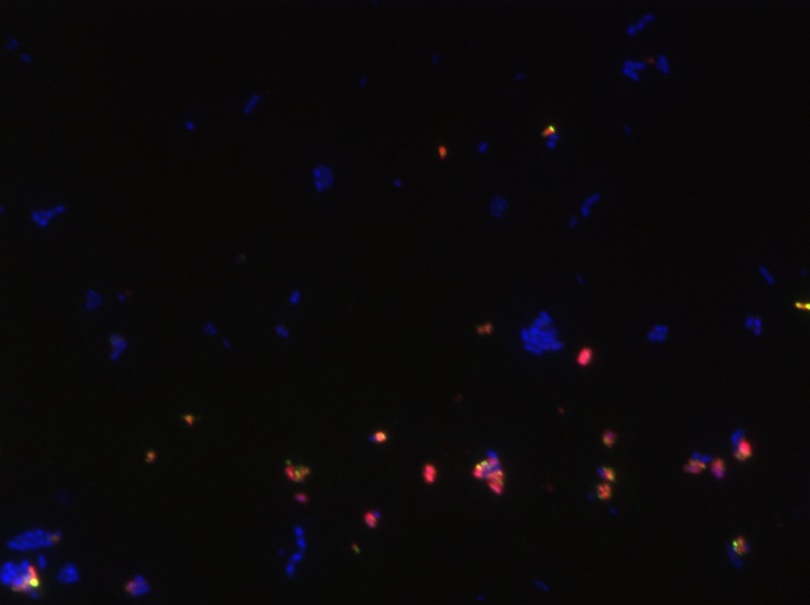
**

**Supplementary Fig. S2** Fluorescence in situ hybridization (FISH) for the co-cultures using a bacterial 16S rRNA-targeted probe (labeled by Alexa Fluor 594, red fluorescence) and an archaeal 16S rRNA-targeted probe (labeled by Alexa Fluor 488, green fluorescence). After hybridization, the cells were counterstained with DAPI (blue fluorescence)


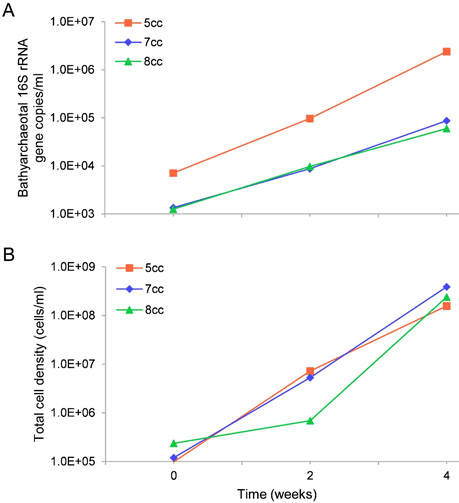


**Supplementary Fig. S3** Bathyarchaeotal 16S rRNA gene copy numbers (**a**) and total cell density (**b**) of the co-culture 5cc, 7cc and 8cc during four weeks of incubation under autotrophic conditions (NaHCO_3_/H_2_)
